# Efficacy of a 6-month supported online programme (Feeling Safer) for the treatment of persecutory delusions: protocol for a randomised controlled trial

**DOI:** 10.1136/bmjopen-2025-104580

**Published:** 2025-06-06

**Authors:** Daniel Freeman, Richard Emsley, Laina Rosebrock, Anthony Morrison, Kate Chapman, Stephanie Common, Robert Dudley, Louise Isham, Thomas Kabir, Alex Kenny, Jason Freeman, Ariane Beckley, Verity Westgate, Natalie Rouse, José Leal, Megan McGovern, Felicity Waite

**Affiliations:** 1 Department of Experimental Psychology, University of Oxford, Oxford, UK; 2 Oxford Health NHS Foundation Trust, Oxford, UK; 3 Department of Biostatistics and Health Informatics, Institute of Psychiatry, Psychology and Neuroscience, King’s College London, London, UK; 4 King’s Clinical Trials Unit, King’s College London, London, UK; 5 The Psychosis Research Unit, Greater Manchester Mental Health NHS Foundation Trust, Manchester, UK; 6 Avon and Wiltshire Mental Health Partnership NHS Trust, Bristol, UK; 7 Tees, Esk and Wear Valleys NHS Foundation Trust, Stockton on Tees, UK; 8 Cumbria, Northumberland, Tyne and Wear NHS Foundation Trust, Newcastle, UK; 9 University of York, York, UK; 10 Department of Psychiatry, University of Oxford, Oxford, UK; 11 McPin Foundation, London, UK; 12 Health Economics Research Centre, Nuffield Department of Primary Care Health Sciences, University of Oxford, Oxford, UK

**Keywords:** MENTAL HEALTH, Randomized Controlled Trial, Schizophrenia & psychotic disorders, Adult psychiatry, Digital Technology

## Abstract

**Introduction:**

Persecutory delusions are very common in severe mental health disorders such as schizophrenia. Existing treatments often do not work well enough. We developed a face-to-face theory-driven psychological intervention, called Feeling Safe, that produces very large reductions in persistent persecutory delusions. The challenge now is to make Feeling Safe widely available. So, we developed a 6-month supported online version, called Feeling Safer. The aim is an intervention that patients can easily access and use, reduces persecutory delusions and can be supported by a range of mental health professionals in less contact time than face-to-face therapy. Initial proof of concept testing of Feeling Safer was very encouraging. In a randomised controlled trial, we now plan to test whether Feeling Safer is efficacious for patients and can be successfully delivered by any of three different mental health staff groups (peer-support workers, graduate psychologists and cognitive behavioural therapy (CBT) therapists). We will also test whether Feeling Safer works equally across gender, age, ethnicity and cognitive functioning (moderation) and whether Feeling Safer works via the targeted psychological processes (mediation).

**Methods and analysis:**

The study design is a multicentre, single-blind (outcome assessor), parallel, four-arm randomised controlled trial; 484 patients with persistent persecutory delusions will be randomised to one of the four conditions (1:1:1:1): Feeling Safer (added to treatment as usual (TAU)) supported by peer-support workers, or Feeling Safer (added to TAU) supported by graduate mental health workers including assistant psychologists, or Feeling Safer (added to TAU) supported by CBT therapists or TAU. Feeling Safer will be provided for 6 months with a staff member. Assessments will be conducted at 0, 3, 6 and 9 months by research assistants blind to group allocation. The primary outcome is severity of persecutory delusions at 6 months rated with the Psychotic Symptoms Rating Scale—Delusions. The secondary outcomes are other psychiatric symptoms (depression, anxiety, insomnia, agoraphobia and paranoia), psychological well-being, recovery, activity and health-related quality of life. Analysis will be conducted under a treatment policy strategy following the intention-to-treat principle, incorporating data from all participants including those who do not complete treatment. Moderation and mediation will be tested. A within-trial cost-effectiveness analysis will be conducted of Feeling Safer compared with TAU.

**Ethics and dissemination:**

The trial has received ethical approval from the NHS Health Research Authority (23/LO/0951). Informed consent will be obtained from all participants. A key output will be an open-access publication in a peer-reviewed journal reporting on the clinical effectiveness of a high-quality supported online programme for the treatment of persecutory delusions that has the potential to be used at scale in mental health services.

**Trial registration number:**

ISRCTN93974770.

**STRENGTHS AND LIMITATIONS OF THIS STUDY**

- A supported online programme (Feeling Safer) is a means to provide access to evidence-based efficacious psychological therapy to many more people with psychosis.
- This is a four-arm randomised controlled trial that will provide separate estimates of the treatment effects of Feeling Safer when it is supported by each of three different mental health staff groups.
- Moderation, mediation and cost-effectiveness analyses are built into the trial design.
- The trial will not be able to determine the components of Feeling Safer that lead to clinical benefits.
- The trial is only powered to detect moderate improvements with treatment in persecutory delusions.

## Introduction

 Despite current service provision, many patients diagnosed with (non-affective) psychosis (eg, schizophrenia) still experience persecutory delusions. Patients have strongly held beliefs such as ‘When I go out the devil and others persecute me’, ‘People are trying to cause me physical, mental, and emotional harm’, ‘People know what I’m thinking and want to kill me’, ‘People see me as an easy target and do things to belittle me’ and ‘Someone intends to kill me’. Feeling so unsafe leads to withdrawal from activities, which adversely affects physical and mental health. Half of patients with persistent persecutory delusions have levels of psychological well-being in the bottom 2.5% of the general population,^1^ three-quarters of patients have suicidal ideation^2^ and the delusions can directly lead to hospital admission.^3^ Carers describe how these delusions can fracture relationships.^4^

Translating from our empirically established theoretical model,^5^ we carefully developed a new in-person individual cognitive treatment designed to produce large effect size reductions in persistent persecutory delusions. In our theoretical model, persecutory delusions are conceptualised as inaccurate threat beliefs developed in the context of genetic and environmental risk. These beliefs are maintained by a number of psychological processes including excessive worry, low self-confidence, poor sleep, anomalous experiences such as dissociation and hearing voices and defence behaviours. The clinical implication of the model is that safety must be relearned. This is achieved by entering feared situations after having systematically weakened the influence of the maintenance factors. We carried out a series of studies evaluating a number of individual modular elements of the intervention, for example targeting worry, sleep, self-confidence and defence behaviours. As an illustration, in a randomised controlled clinical trial with 150 patients with persecutory delusions, we showed that our six-session worry intervention delivered over 6 weeks led to reductions in both levels of worry (Cohen’s d=0.5) and delusions (Cohen’s d=0.5) that were maintained at 6-month follow-up.^6^ A mediation analysis showed that the changes in worry explained the changes in delusions. Treatment effects were not moderated by severity of delusions, intellectual functioning, illicit drug use or cognitive functioning.

The modules were then combined into the Feeling Safe programme, which is personalised and includes patient preference. The design principles underpinning Feeling Safe have been described elsewhere.^7^ An initial proof-of-concept test indicated the potential for a very large treatment effect.^8^ We then reported the results of a randomised controlled trial (RCT) with 130 National Health Service (NHS) patients with persistent persecutory delusions in the context of non-affective psychosis.^9^ A group of people with lived experience of psychosis advised on the conduct of the trial throughout. The form of the trial was intentionally tough: we compared the Feeling Safe programme to an alternative psychological approach (befriending) provided by the same therapists over the same time period. In this way, we could tell if the Feeling Safe programme brings benefits beyond those that come with a positive therapeutic relationship. The Feeling Safe programme led to large reductions in persecutory delusions, even above the alternative psychological therapy. The effect size (Cohen’s d) at the end of treatment above the alternative therapy was 1.2 for delusion severity, and at a 12-month follow-up, the effect size benefit for delusion severity above the alternative treatment was 0.9 (ie, there were large additional benefits for patients over befriending that were maintained over time). Patients were also significantly happier after Feeling Safe: their psychological well-being improved even above the benefits of befriending. A parallel peer-method qualitative study also demonstrated the strong appeal of Feeling Safe to patients.^10^ For example, patients reported: ‘I was able to go out that bit more and try and lead as much of an ordinary life as possible’, ‘I’m much more outgoing than I was now. I’m, sort of, on the up in a way. I’ve got a job and I’m applying for lab jobs and things like that and all those things make you feel good about yourself’, and ‘I guess the Feeling Safe study taught me that the things that you fear the most, you have to do and to get over them and that’s just the way it is’.

Feeling Safe is the most effective psychological intervention for persecutory delusions. It provides new optimism in the treatment of delusions. The problem to be addressed now is how to get this highly effective treatment to as many patients as need it. Feeling Safe was delivered by clinical psychologists in an average of 20 hours of in-person individual sessions over 6 months. An average of six and a half sessions was spent outside for behavioural tests. Of the remaining sessions, approximately half were home visits and half were appointments in mental health clinics. There were no remote sessions. This is not a model of delivery that can be scaled up to the degree necessary, due to a limited number of therapists.

To overcome this problem, we have developed a 6-month guided online version called Feeling Safer. We wanted a programme that could be supported by a range of staff groups; largely be delivered remotely to minimise the considerable time often spent travelling to patient homes; lessen the overall therapist time; expand the content coverage of the intervention and reduce the likelihood of therapist drift during intervention delivery. To reflect the variability in the patient group, we allowed flexibility in the level and type of support provided by the mental health staff member. Though the programme was principally to be guided via brief weekly remote meetings between patient and mental health staff member, a limited number of in-person meetings could be held (ie, we envisaged an optional degree of blended treatment). In proof-of-concept testing with 14 patients with persistent persecutory delusions, satisfaction and usability ratings of the programme were high, there were very large reductions in the delusions, and therapist time was substantially reduced.^11^ The results met an a priori decision criterion for progression to an RCT. We have also recently elaborated on a counter-weight model of persecutory delusions that guides Feeling Safe and Feeling Safer’s therapeutic targets and approach.^12^

### Research questions

#### Primary research question

For patients with persistent persecutory delusions in the context of a psychosis diagnosis, can Feeling Safer, when added to treatment as usual (TAU), delivered by either peer-support workers (PSW), graduate mental health workers or cognitive behavioural therapy (CBT) therapists, compared with TAU, reduce persecutory delusions? The primary end-point is 6 months (end of therapy).

#### Secondary research questions

##### Outcomes

1. Compared with TAU, does Feeling Safer lead to improvement in other psychiatric symptoms (depression, anxiety, insomnia, agoraphobia and paranoia), psychological well-being, recovery, activity and health-related quality of life?
2. Are Feeling Safer treatment benefits maintained at the later follow-up (9 months)?

##### Moderation

1. Are Feeling Safer treatment outcomes moderated by age, ethnicity, gender or cognitive functioning?

##### Mediation

1. Do changes in the targeted psychological factors (safety beliefs, vulnerability beliefs, negative and positive self-beliefs, positive other-beliefs, worry, insomnia, defence behaviours, anomalous experiences and social support) mediate change in delusions with Feeling Safer?

We will also conduct a health economic evaluation of Feeling Safer and a separate qualitative evaluation.

## Methods and analysis

### Trial design and flowchart

**Figure 1:**
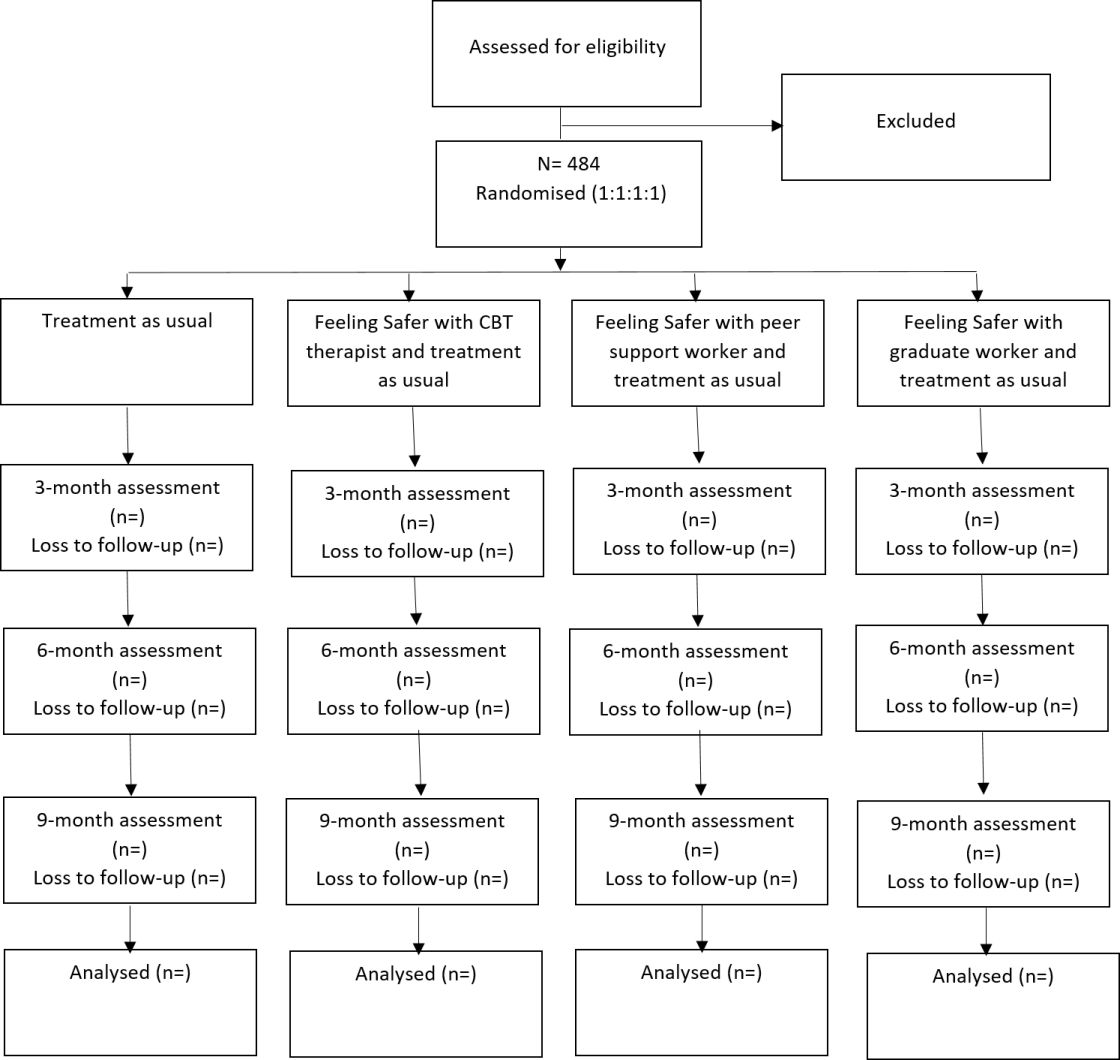
Trial flow diagram. CBT, cognitive behavioural therapy.

The design is a multicentre, single-blind (outcome assessor), parallel, four-arm RCT testing the addition to TAU of Feeling Safer provided by each of three staff delivery groups against TAU. Moderation and mediation tests are built into the trial design. We wish to determine the ‘in toto’ benefits of the programme when added to standard care (ie, the total effect for patients if implemented in the NHS). 484 patients will be randomised to one of the four conditions: Feeling Safer (added to TAU) supported by PSW, or graduate mental health workers including assistant psychologists, or CBT therapists or TAU. Feeling Safer will be provided for 6 months with a staff member. Assessments will be conducted at 0, 3, 6 (end of treatment) and 9 (follow-up) months in person or online by research assistants blind to group allocation. A summary of the trial design can be seen in figure 1. The trial will take place in five centres: Bristol/Darlington/Manchester/Newcastle/Oxford. The NHS trusts will be Avon and Wiltshire Mental Health Partnership NHS Trust; Greater Manchester Mental Health NHS Foundation Trust; Tees, Esk and Wear Valley NHS Trust; Cumbria, Northumberland, Tyne and Wear NHS Foundation Trust; Oxford Health NHS Foundation Trust; Berkshire Healthcare NHS Foundation Trust; Northamptonshire Healthcare NHS Foundation Trust; Central and North West London NHS Foundation Trust (Milton Keynes). The University of Oxford is the trial sponsor. There is a data monitoring and ethics committee (DMEC) and trial steering committee.

### Randomisation, blinding and code-breaking

Patients will be randomised once they have completed the baseline assessment. Randomisation, using an online system from King’s Clinical Trial Unit (CTU), will use a permuted blocks algorithm, with randomly varying block size, stratified by centre, in a 1:1:1:1 ratio.

The research assessors will be blinded to group allocation, but the patients and allocated staff member will not be (they cannot be blinded to whether a psychological intervention is delivered or not). The Feeling Safer treatment deliverers will inform patients of the randomisation outcome to ensure that the research assessors remain blinded to group allocation. Precautionary strategies to prevent unblinding of allocation include the staff member and assessor considering room use and booking arrangements; patients being reminded by the assessor not to talk about their allocation result; and, after the initial assessment, the assessor not looking at the patient’s clinical notes. If an allocation is revealed between assessment sessions, this is logged by the trial coordinator, and whenever possible, re-blinding will occur using another assessor.

### Participants

The principal method of recruitment will be via seeking referrals to the trial from the relevant clinical teams in the participating mental health trusts (eg, adult community mental health teams, early intervention services, and inpatient units). Patients interested in taking part will be approached by the research team with the approval of the clinical team, given information about the trial (including a Patient Information Sheet) (see online supplemental materials) and eligibility screening conducted. All suitable patients will be given at least 24 hours to consider taking part in the trial, although in practice, it is typically a week. Our Lived Experience Advisory Panel (LEAP) has also emphasised the importance of patients in participating trusts being able to self-refer to the trial. This will minimise the chances that patients are overlooked by clinical teams or because their clinician was not present at a referral meeting. Hence, we will also advertise the study to patients via posters and leaflets in NHS buildings. However, in all instances, we will seek to confirm that a member of the clinical team gives approval for a patient to enter the trial and to complete the necessary check of eligibility and risk status. Written informed consent will be obtained from all participants (see online supplemental materials).

The inclusion criteria are as follows: participant is willing and able to give informed consent for participation in the trial; aged 16 years or older; attending NHS mental health services for the treatment of psychosis; persistent (at least 3 months) persecutory delusion (as defined by Freeman and Garety^13^), held with at least 50% conviction and no planned significant medication changes at the outset of participation.

The exclusion criteria are as follows: a primary diagnosis of another mental health condition (eg, substance use disorder) that would be the first clinical priority to treat; current engagement in any other intensive individual psychological therapy or a significant change in medication; in forensic settings or Psychiatric Intensive Care Unit; command of spoken English inadequate for engaging in the therapy or significant learning difficulties that would prevent the completion of assessments or the therapy. A participant may also not enter the trial if there is another factor (eg, current active suicidal plans that need to be the focus of intervention), which, in the judgement of the investigator, would preclude the participant from providing informed consent or from safely engaging with the trial procedures. Reason for exclusions will be recorded.

### Assessments

The primary outcome is the severity of persecutory delusion assessed by the Psychotic Symptoms Rating Scale (PSYRATS)—Delusions.^14^

Secondary outcomes are as follows: depression (PHQ-9);^15^ anxiety (GAD-7);^16^ insomnia (eight-item Sleep Condition Indicator);^17^ agoraphobia (Oxford Agoraphobic Avoidance Scale);^18^ paranoia (Revised Green *et al* Paranoid Thoughts Scale);^19^ psychological well-being (Warwick-Edinburgh Mental Well-being Scale);^20^ personal recovery (the Questionnaire about the Process of Recovery—15 items);^21 22^ meaningful activity (time budget)^23^ and quality of life (EQ-5D-5L and ReQol-20).^24 25^

The moderators are as follows: age, gender, ethnicity and working memory (WAIS-III digit span forward, digit span backward and letter-number sequencing).^26^

The mediators are as follows: vulnerability and safety beliefs;^9^ negative self-beliefs and positive-other beliefs (Brief Core Schema Scale—negative self and positive others);^27^ positive self-beliefs (Oxford Positive Self Scale Short Form);^28^ worry (Dunn Worry Questionnaire);^29^ insomnia (Sleep Condition Indicator);^17^ defence behaviours (Oxford Paranoia Defence Behaviours Questionnaire),^30^ anomalous experiences (Specific Psychotic Experiences Questionnaire—hallucinations^31^ and Global Felt Sense of Anomaly scale (GFSA))^32^ and social support (Multidimensional Scale of Perceived Social Support).^33^

Service use data will be collected with an adapted version of the Client Service Receipt Inventory (CSRI).^34 35^

For patients in the therapy arms, there will be non-blinded collection by the treatment deliverers of a Modified Client Satisfaction Questionnaire^36 37^ and the theoretical framework of acceptability questionnaire.^38^

### Feeling Safer: the supported online programme

Feeling Safer is a guided online progressive web app recommended for adults (16 years or older) attending psychosis services who have a persecutory delusion. The software is intended to reduce persecutory delusions. Feeling Safer is a UKCA marked, class I medical device (standalone software as a medical device). It is available on all screen sizes, from desktops to mobile phones. Feeling Safer is standalone and not connected to NHS records. The treatment was programmed by a company that is ISO27001 and Cyber Essentials Plus accredited (ISO27001 is an international standard for the management of information security; Cyber Essentials is a UK government-backed set of standards for online security.) Both the design and development work to build the application were guided by AA Web accessibility standards, following industry best practice. Feeling Safer has been designed to meet the requirements of the Organisation for the Review of Care and Health Apps (ORCHA).

The programme has separate components for patients, deliverers and system administrators. The Feeling Safer application allows patients to complete an assessment and then work through up to 10 digitised Feeling Safer modules. A mental health professional portal allows staff to view information about their patients’ use of the programme (eg, sessions completed, questionnaire scores). Finally, an administrator portal allows administrators to manage the professionals that may access the mental health professional portal. The application requires existing users to be logged in before they can interact with most of its functionality, leaving only selected pages to be accessed publicly (such as the Login, Privacy and Instructions for Use pages). Only authorised staff can create a new user.

The patient experience follows the logic of the original Feeling Safe intervention. They begin with an introductory module, which provides information about the therapy together with animations featuring patients recounting their personal stories. Patients then complete an assessment to provide the relevant treatment modules. The intervention is thus personalised. The presentation of the modules includes general recommendations for order of completion. The patient then works through a module of their choosing; once complete, they go on to the next relevant module. Throughout, there are regular assessments, with information on progress fed back graphically to the user. Module content is conveyed by text and (optionally) voice; animations and videos. Each module has an introductory animation (voiced by DF) and then includes animations featuring patient voices. Calendar functionality enables patients to schedule, and be reminded of, offline homework tasks. A ‘bad day’ section provides guidance for when people are especially struggling and finding it too difficult to engage with session content. And there is also a private diary section (with audio or written input) where patients can keep notes. It was expected for patients to log in two or three times a week.

All modules currently comprising Feeling Safe were included, with the content substantially rewritten: getting better sleep; winning against worry; boosting self-confidence; feeling safe with voices and finding safety. Five new modules were added: dealing with bad memories; getting active; finding balance (emotional regulation); getting the fears out (emotional expression^39^) and connecting with other people. Each module is broken down into many 10–15 min sessions. These sessions include tasks to complete offline. The underpinning therapeutic approach is cognitive-behavioural.

It is expected that the mental health staff member supporting Feeling Safer meets in person with the patient at the beginning to explain the programme, provide the access link and check that the person is able to log in. This meeting is also important for developing a therapeutic alliance. Regular check-ins or meetings, typically weekly, with the mental health staff member are expected. These are conducted remotely (eg, via telephone or video call). A smaller number of in-person sessions may be provided. These should typically be used for the staff member to help the person return to everyday activities or for behavioural tests to learn safety. The level—and type—of staff support can be tailored to a patient’s need. The staff-supported provision of Feeling Safer takes place over 6 months. Patients can still access the programme after this period but without staff support. If a patient does not have a suitable device to access Feeling Safer, this is provided for them. The staff member supporting Feeling Safer is expected to have weekly clinical supervision.

### Control condition

Participants who are allocated to the control arm will continue to receive their usual care (TAU). No additional interventions will be offered by the research team. TAU for the participants within this trial will vary but typically consist of prescription of psychiatric medications and meetings with a mental health practitioner. We will collect detailed data on TAU.

### Serious adverse events (SAEs)

SAEs are recorded using a Serious Adverse Event (SAE) Report Form. Each participant’s medical notes will be systematically checked for SAEs following completion of the final assessment to ensure that all SAEs are recorded. We will also record any SAEs that come to the attention of the research team. An adverse event is defined by the ISO14155:2011 guidelines for medical device trials as serious if it: (A) results in death, (B) is a life-threatening illness or injury, (C) requires hospitalisation or prolongation of existing hospitalisation, (D) results in persistent or significant disability or incapacity, (E) medical or surgical intervention is required to prevent any of the above, (F) leads to fetal distress, fetal death or consists of a congenital anomaly or birth defect or (G) is otherwise considered medically significant by the investigator. Life-threatening in the definition of an SAE refers to an event in which the subject was at risk of death at the time of the event; it does not refer to an event that hypothetically might have caused death if it were more severe. A planned hospitalisation for a pre-existing condition, without a serious deterioration in health, is not considered to be an SAE.

It is relatively common for this patient group to have SAEs, typically psychiatric hospital admissions, physical health hospital admissions and suicide attempts.

The relationship between Feeling Safer or other research procedures and the occurrence of each SAE will be assessed and categorised. The chief investigator will use clinical judgement to make an initial assessment of the relationship. Alternative causes, such as the natural history of the participant’s underlying condition, concomitant therapy, other risk factors, etc, will be considered. The investigator will also consult the current version of the risk analysis report. The chief investigator will make an initial assessment of whether the SAE is potentially related to the device or trial procedures, and the expectedness, and report as necessary to the regulatory authorities within the appropriate timescales (eg, related and unexpected SAEs to the research ethics committee and adverse incidents to the Medicines and Healthcare products Regulatory Agency (MHRA)). The decisions about relatedness and expectedness will be reviewed by the DMEC chair (an independent clinician) in the first instance, and later taken to a DMEC meeting.

We will also record adverse events that are not serious. This would include any adverse device effects from the online programme, including those resulting from insufficient or inadequate instructions for use, deployment, installation or operation, or any malfunction of the software. It also includes any event resulting from user error or intentional misuse.

### Data management

All trial data are entered on paper or electronic clinical research forms (CRFs) and transcribed or entered directly to the clinical data management system. The web-based MACRO Electronic Data Capture (EDC) system is used, programmed and managed by the King’s CTU. Data are pseudonymised using a unique study ID. Personal data and participant identification codes are kept separately from the research data. Access to these data is strictly on a need to know basis. Data are transferred from paper CRFs to the clinical database, or recorded directly on eCRFs as soon as possible after a study visit. Validation of all data entered into the clinical database is achieved through manual review. All critical data items will be 100% checked against original source documents, where applicable, to ensure accuracy, and an error rate is established across all fields to ensure a consistently accurate data set.

### Analysis

A full statistical analysis plan will be approved before any analysis. We will report data in line with the Consolidated Standards of Reporting Trials (CONSORT) 2018 SPI^40^ and 2025 statements^41^ showing attrition rates and loss to follow-up. All outcome analyses will be conducted by statisticians at King’s College London under King’s Clinical Trials Unit standard operating procedures. The target estimand is the treatment policy estimand, and all primary and secondary analyses will be carried out following the intention to treat principle, incorporating data from all randomised participants who provide follow-up data for at least one timepoint, irrespective of intervention received. Every effort will be made to follow up all participants in both arms for research assessments.

Descriptive statistics within each randomised group will be presented for baseline values. These will include counts and percentages for binary and categorical variables, and means and SD or medians with lower and upper quartiles, for continuous variables, along with minimum and maximum values and counts of missing values. There will be no tests of statistical significance or CIs for differences between randomised groups on any baseline variable.

Treatment effects for the primary and secondary outcomes will be estimated using linear mixed models fitted to outcome variables at all follow-up timepoints. Fixed effects will be centre, baseline assessment for the outcome under investigation, treatment, time and time*treatment interactions. Participants will be included as a random intercept to account for repeated measures and Feeling Safer deliverer to account for clustering by Feeling Safer deliverer, treating the control participants as clusters of size 1. Marginal treatment effects will be estimated for the primary outcome (PSYRATS-Delusions) at each time point and reported separately as adjusted mean differences in scores between the groups with 99% CIs and two-sided p-values. For secondary outcomes, the same approach will be followed using linear mixed models to estimate and report the treatment effect at each time point. Cohen’s d effect sizes will be calculated as the adjusted mean difference of the outcome divided by the sample SD of the outcome at baseline. These will be displayed in a forest plot showing the treatment effects on the primary and the secondary outcomes at 6 months. We will also assess minimal response rates, defined as a 20% reduction in PSYRATS-Delusions total score.

We will check for differential predictors of missing outcomes by comparing responders to non-responders on key baseline variables. Any significant predictors will be included in the analysis models in a sensitivity analysis. This accounts for missing outcome data under a missing at random assumption, conditional on the covariates included in the model. As a sensitivity analysis, we will assess whether treatment adherence is associated with missing data, and if it is associated, use inverse probability weights or multiple imputation to compare results.

To test the moderation hypotheses, we will extend the analysis model for the PSYRATS-Delusions to include as fixed effects the putative moderator and its interaction with treatment; the coefficient of the interaction tests whether there is a differential treatment effect across levels of the moderator variable.

To test the mediation hypotheses, we will estimate causal mediation estimands using parametric regression models/structural equation models that are extended for multiarm trials and allow for multiple mediation. All mediation analyses will be adjusted for baseline measures of the mediator, outcomes and possible measured confounders.

A separate Health Economic Analysis Plan will be written in accordance with best practice for economic evaluations alongside a clinical trial. We will conduct a within-trial cost-utility analysis following the intention-to-treat principle and considering two perspectives: (1) NHS and Personal Social Services (NHS&PSS) and (2) societal (incorporating NHS&PSS and wider costs). The primary outcome measure of the economic evaluation will be incremental cost per quality-adjusted life year (QALY). The health-related quality of life instruments EQ-5D-5L and ReQoL-20 collected at baseline, 6 and 9 months will provide the utility values for the calculation of QALYs. We will estimate the costs of providing the Feeling Safer intervention using a micro-costing approach capturing training, travel and therapy delivery time, supervision time, technical support staff, consumables and capital expenditures. An adapted version of the CSRI^34 35^ will be used to collect participant data on use of healthcare (eg, GP attendances, A&E attendances, contacts with mental health teams and admissions to acute hospital) and social care services (eg, social worker, recovery worker), health-related impact on work, contact with criminal justice services and informal care received. Data on admissions to psychiatric hospital and psychotropic medication prescribed will be obtained by reviewing mental health trust medical record notes. These will be valued using national unit costs data sets such as NHS Reference Costs, British National Formulary and published literature, where appropriate. Costs and QALYs will not be discounted given the 12 months horizon. Differences in costs and utilities between arms will be estimated using multilevel mixed-effects linear regression models to allow for multiple follow-ups clustered by participant. Missing data will be imputed following best practice methods, and joint uncertainty around incremental total costs and QALYs will be estimated using seemingly unrelated regression.

The RCT is powered to test whether treatment delivery of Feeling Safer is effective for each staff group compared with TAU (not to test equivalence or non-inferiority between staff groups). We will account for multiple testing of three comparisons (PSW vs TAU; graduate mental health worker vs TAU; CBT therapist vs TAU), so the conservative alpha level is 0.05/3=0.0167. For 90% power, allowing for 10% attrition, we require 121 people per arm to detect an effect size of 0.5 on the PSYRATS total score (ie, a moderate effect size).^42^ The face-to-face Feeling Safe programme produced a large effect size (d=1.2) reduction in PSYRATS total score above that of an alternative (and beneficial) psychological therapy.^9^ The within-group effect of Feeling Safe on PSYRATS total scores was extremely large (d=2.8), which was even higher than in our original proof-of-concept testing (d=2.3).^8^ It is a therapy deliberately designed to produce large effects. Nonetheless, to be conservative, we power the trial of the supported online version (Feeling Safer) to detect a moderate effect (d=0.5), just above that of general CBT for psychosis approaches compared with TAU (d=0.3).^43^ Minimal response is defined in schizophrenia trials as a 20% symptom reduction.^44^ The trial will have 98% power to detect Feeling Safer achieving a minimal response (20% reduction in total PSYRATS score) in 60% of patients compared with 30% in TAU and 90% power to detect Feeling Safer achieving a minimal response in 55% of patients compared with 30% in TAU.

### Patient and public involvement

The study team has two individuals with lived experience leading PPI (TK and AK). The study is supported by a LEAP made up of approximately a dozen people with experience of psychosis who live in the sites taking part in the trial. This group will meet at regular intervals during the trial. We also set up a UK-wide Involvement Network for Feeling Safer. The network comprises 30 people with relevant lived experience and includes diversity in age, gender, ethnicity and location. We also engaged with several community groups. To date, 67 people with lived or caring experience of psychosis have contributed just over 400 hours of input. At the start of the project, seven in-person group meetings were held across the country to gain insights into the potential opportunities and challenges in developing a guided online programme. Thirty-nine people with lived experience carried out a line-by-line review of all module content. Feedback was provided in written form and in 10 group meetings. There was also ad hoc consultation on elements of the programme. Iterative user testing took place during development. A final usability testing session with the completed programme was conducted with six lived experience advisors, with excellent ratings. The LEAP has also reviewed and advised on all study materials (eg, the Patient Information Sheet, leaflet and poster).

## Ethics and dissemination

The trial has received Health Research Authority (HRA) approval (IRAS 330744). The trial received ethical approval from the NHS London—Harrow Research Ethics Committee (23/LO/0951). Informed consent will be obtained from all participants. Participants are currently being recruited into the trial. The first randomisation was conducted on 22 January 2025. Any changes to the trial protocol will have sponsor and ethical approvals. It is expected that the study will be completed by May 2027. The results of the trial will be published in a peer-reviewed journal and made open access. Deidentified participant data will be available in anonymised form on reasonable request, subject to review and contract with the University of Oxford, following the publication of results.

## Supplementary material

10.1136/bmjopen-2025-104580 online supplemental file 1

10.1136/bmjopen-2025-104580 online supplemental file 2
