# Supplementary material for: Efficacy of a 6-month supported online programme (Feeling Safer) for the treatment of persecutory delusions: protocol for a randomised controlled trial
Source: BMJ Open. 2025 Jun 6;15(6):e104580. doi: 10.1136/bmjopen-2025-104580 (PMC12161337; doi:10.1136/bmjopen-2025-104580)
Supplement: online supplemental file 1 [file bmjopen-15-6-s001.docx]

**
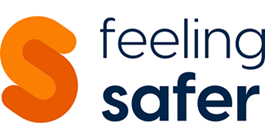
**

**Feeling Safer**

**Participant Information Sheet**

We would like to invite you to take part in our study of whether a new therapy is helpful. Before you decide, we would like you to understand why the research is being done and what it would involve for you. One of our team will go through this information sheet with you and answer any questions you have. Please ask us if anything is unclear or you would like more information.

# Key facts:

- Feeling Safer is for people attending mental health services, aged 16 or older, who are feeling very worried or concerned that others are trying to harm them.
- Feeling Safer is a new six-month programme designed to help people feel safer, happier, and get back to doing more of the things they want to be doing. It is an online programme (accessed via a computer, tablet or smart phone) that is guided by a staff member. If you do not have a suitable device one can be provided.
- Feeling Safer involves logging onto the programme, typically 2-3 times a week for 20 minutes, and completing a range of tasks. You get to choose the tasks that you want to do. They could include, for example, how to feel more self-confident, worry less, sleep better, find out how safe you are, or to be more active. There is a weekly check-in by phone or video call with a staff member and also some face-to-face meetings.
- The purpose of this research is to see whether Feeling Safer may be helpful for people. To do this, three-quarters of participants will receive Feeling Safer (in addition to their usual care) and one-quarter of the participants will simply continue with their usual care. Whether a person has Feeling Safer will be decided by chance. We then compare how people who have had Feeling Safer have got on compared to people who have not had the therapy.
- If you do receive Feeling Safer then the staff member may be either a peer support worker, assistant psychologist (or mental health and wellbeing practitioner), or CBT therapist. The type of staff member who supports you with Feeling Safer is decided by chance.
- Everybody will also meet a research assistant at the beginning and then after 3, 6, and 9 months. This is to carry out assessments to see how people get on. You will be compensated for your time completing these assessments.
- It is entirely your choice whether to take part in the research or not. Your usual treatment will not be affected in any way by your decision and you will continue to have access to the standard of care you currently have. Even if you decide to take part, you will be free to leave at any time, for any reason.

# What is the purpose of the Feeling Safer trial?

Many people worry that others are trying to harm them in some way. It can be hard to trust. The world can feel unsafe. We have developed a six-month online programme, supported by a mental health staff member, called Feeling Safer. Feeling Safer is designed to help people feel safer, happier, and get back to doing more of the things that they want to be doing. The purpose of the research study is to see whether Feeling Safer is potentially helpful for people.

# What is the new Feeling Safer therapy?

Feeling Safer has been developed following fifteen years of research. It is an online programme available to people via computer, tablet, or smartphone. If you don’t have a device then we can provide a smartphone for the duration of the study. There is a separate weekly check-in by telephone or video call with a Feeling Safer staff member. In the online programme there are questions to help determine the information and therapy techniques that may be most suitable for you. You select the ones to complete. The sorts of areas that might be covered include self-confidence, sleep, worry, voices, memories, finding safety, and activities. It is all designed to help people find a greater degree of safety in their lives. It is expected that a person will log on 2-3 times a week for about 20 minutes each time. Your usual NHS care will continue as before. Feeling Safer has been safely and successfully tested in an initial research study with 15 people.

The staff member who supports the use of Feeling Safer will either be a peer support worker (someone who has direct experience of the concerns that Feeling Safer is designed to help with), an assistant psychologist (or mental health and wellbeing practitioner), or a cognitive-behavioural (CBT) therapist. The type of staff member that supports a person with Feeling Safer will be decided by chance, so we can check that the therapy works well when delivered by each type of staff member.

# Who can take part in the research?

The research is for adults (16 years+) who feel very concerned that others are trying to harm them in some way, and are attending mental health services for a diagnosis of psychosis. Excessive fear about others trying to cause harm can have many names. In psychiatric settings it can be known as paranoia or persecutory delusions. Our focus is on people who are worried about harm and want to feel safer. We acknowledge that many people have had very difficult experiences with other people. We will first check with you whether the research study is suitable for you. Our aim is for 484 people to take part in the research.

# Do I have to take part?

No. If you are eligible to take part, it is up to you to decide whether to take part. If you agree to take part we will then give you a consent form to sign. You are free to withdraw at any time, without giving a reason. Your usual treatment will not be affected if you withdraw from this study.

# What will happen if I take part?

Everyone who takes part will be asked to meet with a research assistant four different times for an assessment. The assessments are at the beginning, then after 3 months, 6 months, and 9 months. The meetings will typically take place at your local mental health clinic, or online, or we may be able to meet at your home if you would prefer. At the assessments you’ll also be asked to complete questionnaires on, for example, your confidence, mood, and activities. We expect the assessments to take about ninety minutes each. They can be broken down into smaller chunks if you would like. We will also try to do them at times and locations that are most convenient to you.

After the first assessment, whether or not you have Feeling Safer will be randomly decided by a computer (rather like flipping a coin). This means that you may - or may not - receive Feeling Safer, which is decided by chance. Three out of four people (i.e. three-quarters) who enter the trial will receive Feeling Safer. Everyone will also continue with their usual care from mental health services.

Participation in the trial lasts 9 months in total.

For more information on what participation in the study would look like, please see the diagram below:

Participation lasts about 9 months in total

As part of the study, we would also want to look at your medical notes. The staff member who helps to support you with Feeling Safer will need to do this as part of standard good care in the NHS. The study team will look at your notes to see how you are getting along and to look at the care you receive. They will also record information on the services and support you receive from your medical notes. All of this will be done on a confidential basis.

# Expenses and payment

You will receive £15 for each of the four assessment sessions you take part in, which will be at the beginning of the study and then at 3, 6, and 9 months. If you complete all four assessment sessions this will add up to £60. We will also reimburse you for any reasonable travel costs for attending the assessments.

# What are the possible risks of taking part?

We do not anticipate any major risks from taking part. Those randomised to Feeling Safer can complete the online programme at their own pace in their own time over six months. We have designed the new Feeling Safer therapy to minimise any risks. However, we will check whether there are any potential problems over the course of this research study. People can stop using the programme if they wish. Everyone will have the research assessments, and if these are experienced as upsetting then it is possible to reduce the number of these. It is also possible to stop the assessment or decline to do more assessments. Therefore it is possible to withdraw from the online programme and/or the assessments.

# What are the possible benefits of taking part?

At present we cannot say that taking part in the study will benefit you. We hope that using Feeling Safer will help people feel safer, happier, and to be more active. The research aims to find out whether this is the case.

# Will my taking part in the study be kept confidential?

The study team will keep your name, NHS number, and contact details confidential. To protect your identity, we will not use your name on any of the study documents. Instead of your name, we will assign you a unique code so that your information is kept private.

Your clinical team will be told you are taking part in the trial. All other information from the research assessments will be kept confidential unless you would like your clinical team to know. The exceptions to this are where there are significant concerns about a risk to you or other people. If a member of our team has such concerns we will need to tell your clinical team.

Responsible members of the University of Oxford, <and> regulatory authorities <and the relevant NHS Trust(s)> may be given access to data for monitoring and/or audit of the study to ensure that the research is complying with applicable regulations.

# What will happen to my data?

Data protection regulation requires that we state the legal basis for processing information about you. In the case of research, this is ‘a task in the public interest.’ The University of Oxford, based in the UK, is the data controller and is responsible for looking after your information and using it properly. We will be using information from you and your medical records in order to carry out this study.

Information that can be used to identify you (e.g. your name or NHS number) helps the research team to keep in contact with you about the research study, make sure that relevant information about the study is recorded for your care, and ensures the quality of the study. During the research, the research team will need to use this type of information, but we will try to keep this to a minimum. We will not share this information with anyone outside the research team. We will keep information that might identify you for 3-6 months after the study has finished. This excludes any research documents with personal information, such as consent forms, which will be stored at the University for 10 years after the end of the study.

Your data will be stored securely with our research team at the University of Oxford. Any data that you provide on hard copies (i.e. pen and paper) will be kept in a locked filing cabinet at the University of Oxford. Any electronic data that is collected (e.g. emails, questionnaire scores) will be kept on a computer at the University of Oxford and will be protected with a password. Only members of the research team will know the password or have access to the filing cabinet. Outcome data will also be entered into the secure database managed by the King’s Clinical Trials Unit (King’s College London). Only members of the research team will have access to this database. There may also be short term storage of data during the trial at your local NHS trust, which will be stored in the same secure way.

Your NHS Trust will use your name, NHS number, home address, and contact details to contact you about the research study, and to oversee the quality of the study. They will keep identifiable information about you from this study in keeping with local policy for medical notes retention.

It is possible that in the future other researchers may be interested in carrying out further analysis of group data from the trial. They would only be given group data that has had identifying information about individuals removed (called de-identified data). This means that de-identified data could be shared and reused for scientific purposes to maximise what is learned from the research. Other researchers would not be given any information that could identify individuals.

Data protection regulation provides you with control over your personal data and how it is used. When you agree to your information being used in research, however, some of those rights may be limited in order for the research to be reliable and accurate. Further information about your wishes with respect to your personal data is available at https://compliance.web.ox.ac.uk/individual-rights. You can find out more about how we use your information by contacting the research team, using the contact details given at the end of this document.

# What will happen if I don't want to carry on with the study?

Participation is voluntary and you may change your mind at any stage. Withdrawing from the study will not affect the care you receive from the NHS. You can withdraw from the study, the assessments or both. You can decide that you do not want us to use any of the data you have provided for future study reports and analysis but we will not be able to remove your data from any reports that we have already produced. The database where we record your data will also keep a record of all data entered into the system. You will only be paid for assessments you attend.

# What will happen to the results of this study?

The results of the study are written up, looking at the total effects of the treatment for all patients, and no one is identified. The results will be presented in scientific papers and in conferences. We will make a summary of the results of the study available for you if you would like that. If you would like to see the results summary, we will keep your contact details so we can share this with you.

# Who is organising and funding the study?

The research is funded by the National Institute for Health and Care Research (NIHR), whose mission is to improve the health and wealth of the nation through research. It is also supported by the NIHR Oxford Health Biomedical Research Centre. The study is sponsored by the University of Oxford.

# What if there is a problem?

The University of Oxford, as sponsor, has appropriate insurance in place in the unlikely event that you suffer any harm as a direct consequence of your participation in this study.

If you wish to complain about any aspect of the study, you should contact Professor Daniel Freeman (contact details below) or you may contact the University of Oxford Research Governance, Ethics & Assurance office (RGEA) by telephone 01865 616480 or email the director of RGEA at [rgea.complaints@admin.ox.ac.uk](mailto:rgea.complaints@admin.ox.ac.uk).

The Patient Advisory Liaison Service (PALS) is a confidential NHS service that can provide you with support for any complaints or queries you may have regarding the care you receive as an NHS patient. PALS is unable to provide information about this research study. If you wish to contact the PALS team please contact <insert relevant NHS site phone number and email from the PALS website>

# Who has reviewed the study?

All research in the NHS is looked at by an independent group of people, called a Research Ethics Committee, to protect participants’ interests. This study has been reviewed and given a favourable opinion by the London – Harrow Research Ethics Committee (Ethics REF 23/LO/0951).

** Please do not hesitate to ask us if you have any questions. It can also be helpful to talk to someone else about whether you’d like to take part **

# CONTACT DETAILS

If you have any questions about this study, please do not hesitate to contact us:

| [insert local RA details] | [insert local trial coordinator details] |
| --- | --- |
| [insert local site lead details] | Professor Daniel Freeman (Overall Trial Lead), University of Oxford and Oxford Health NHS Foundation Trust.  Email: [daniel.freeman@psy.ox.ac.uk](mailto:daniel.freeman@psy.ox.ac.uk)  Telephone number: 01865 613109 |
