# Supplementary material for: Efficacy of a 6-month supported online programme (Feeling Safer) for the treatment of persecutory delusions: protocol for a randomised controlled trial
Source: BMJ Open. 2025 Jun 6;15(6):e104580. doi: 10.1136/bmjopen-2025-104580 (PMC12161337; doi:10.1136/bmjopen-2025-104580)
Supplement: Supplementary file 3 [file bmjopen-2025-104580.reviewer_comments.pdf]

PEER REVIEW HISTORY

BMJ Open publishes all reviews undertaken for accepted manuscripts. Reviewers are asked to complete a checklist review form and are provided with free text boxes to elaborate on their assessment. These free text comments are reproduced below.

ARTICLE DETAILS

Title (Provisional)

Efficacy of a six-month supported online programme (Feeling Safer) for the treatment of persecutory delusions: protocol for a randomised controlled trial

Authors

Freeman, Daniel; Emsley, Richard; Rosebrock, Laina; Morrison, Anthony; Chapman, Kate; Common, Stephanie; Dudley, Robert; Isham, Louise; Kabir, Thomas; Kenny, Alex; Freeman, Jason; Beckley, Ariane; Westgate, Verity; Rouse, Natalie; Leal, José; McGovern, Megan; Waite, Felicity

---

This article was not externally reviewed at BMJ Open. Protocols that have been independently assessed prior to submission to BMJ Open are usually fast-tracked to publication on the grounds that further substantial changes will not be possible. This independent assessment will usually be external, independent review for both a substantial grant award from a non-commercial or government funder and ethics approval.
